# A Combined Transcriptomics and Lipidomics Analysis of Subcutaneous, Epididymal and Mesenteric Adipose Tissue Reveals Marked Functional Differences

**DOI:** 10.1371/journal.pone.0011525

**Published:** 2010-07-12

**Authors:** Robert Caesar, Monia Manieri, Thomas Kelder, Mark Boekschoten, Chris Evelo, Michael Müller, Teake Kooistra, Saverio Cinti, Robert Kleemann, Christian A. Drevon

**Affiliations:** 1 Department of Nutrition, Institute of Basic Medical Sciences, Faculty of Medicine, University of Oslo, Oslo, Norway; 2 Wallenberg Laboratory/Sahlgrenska Center for Cardiovascular and Metabolic Research, University of Gothenburg, Gothenburg, Sweden; 3 Department of Molecular Pathology and Innovative Therapies, School of Medicine, University of Ancona, Ancona, Italy; 4 Department of Bioinformatics, BiGCaT, Maastricht University, Maastricht, The Netherlands; 5 Nutrigenomics Consortium, TI Food & Nutrition, Wageningen, The Netherlands; 6 Nutrition, Metabolism and Genomics Group, Division of Human Nutrition, Wageningen University, Wageningen, The Netherlands; 7 BioSciences, TNO-Quality of Life, Leiden, The Netherlands; University of Parma, Italy

## Abstract

Depot-dependent differences in adipose tissue physiology may reflect specialized functions and local interactions between adipocytes and surrounding tissues. We combined time-resolved microarray analyses of mesenteric- (MWAT), subcutaneous- (SWAT) and epididymal adipose tissue (EWAT) during high-fat feeding of male transgenic ApoE3Leiden mice with histology, targeted lipidomics and biochemical analyses of metabolic pathways to identify differentially regulated processes and site-specific functions. EWAT was found to exhibit physiological zonation. *De novo* lipogenesis in fat proximal to epididymis was stably low, whereas *de novo* lipogenesis distal to epididymis and at other locations was down-regulated in response to high-fat diet. The contents of linoleic acid and α-linolenic acid in EWAT were increased compared to other depots. Expression of the androgen receptor (Ar) was higher in EWAT than in MWAT and SWAT. We suggest that Ar may mediate depot-dependent differences in *de novo* lipogenesis rate and propose that accumulation of linoleic acid and α-linolenic acid in EWAT is favored by testosterone-mediated inhibition of *de novo* lipogenesis and may promote further elongation and desaturation of these polyunsaturated fatty acids during spermatogenesis.

## Introduction

Adipose tissue is an organ with complex biology and important medical implications. In mammals adipose tissue is distributed in numerous depots throughout the body and an increasing number of reports emphasize site-specific physiological properties. Many processes, including fatty acid uptake [1], [2], lipolysis and control of energy metabolism [3]–[6], adipokine secretion [2], [7]–[10] and expression of hormone receptors [11], [12], may differ between adipose depots. During high-fat feeding depot-dependent differences in hyperplasia and hypertrophy are observed [13]. Differences within adipose depots, including fatty acid composition and control of lipolysis [14], have also been described.

Site-dependent differences in adipose tissue physiology have been studied in the context of local, specialized functions where adipokines and/or fatty acids released from adipocytes affect the function of surrounding tissues. An example of this is the interaction with blood vessels where perivascular adipose tissue has been shown to exert a dual regulatory role in modulating vessel function, releasing vasoconstrictive as well as vasorelaxing factors [15], [16]. Another local function attributed to adipose tissue is the paracrine interaction with adjacent lymph nodes [14]. Adipocytes and lymphocytes may communicate via local secretion of cytokines. Importantly, the properties of adipocytes surrounding lymphoid tissue appear to be controlled by paracrine interaction with the adjacent immune cells. Perinodal adipocytes are enriched in polyunsaturated fatty acids (PUFA). Fatty acids are released from adipocytes in response to local lipolytic signals and are incorporated into membrane phospholipids of lymphoid cells. The secreted PUFAs are believed to be utilized as precursors for eicosanoids and docosanoids involved in inflammatory activity [17]–[20]. The role of adipocytes as a local storage of fatty acids for immune cells highlights that fatty acids in adipocytes constitute an important reservoir of biosynthetic building blocks.

Epididymis is another organ closely associated with adipose tissue. It is well established that deficiency of dietary essential fatty acids is associated with reduced fertility, and sperm phospholipids are characterized by high proportions of long-chain PUFA [21]–[25] (Table S1). The high degree of fatty acid unsaturation has been postulated to affect fluidity of sperm cell membranes required for movement and fusion occurring during fertilization [26]. There are also several other mechanisms by which PUFA may exert their biological effects on sperms like acylation of proteins, precursors of eicosanoids and docosanoids, and acting as ligands for transcription factors [27]. Moreover, ω-3 fatty acids increases the activities of β-oxidation and Krebs cycle enzymes in many tissues [28]–[30] and this may also be the case for spermatozoa. This raises the possibility that PUFA enhance sperm mobility by increasing their metabolic activity. The proportion of sperm PUFA has been related to semen quality [24] and particularly the content of docosahexaenoic acid (DHA) has been related to sperm concentration and motility [24], [25], [28].

Despite many reports on diet-induced obesity no investigator has previously performed a global, time-resolved comparison of gene expression in adipose depots during high-fat feeding. In our present study we combine microarray, histology, targeted lipidomics and biochemical analyses of metabolic processes to identify differences between mesenteric, subcutaneous and epididymal adipose tissue. To mimic the way adipose tissue is exposed to fatty acids in humans we used as a model the transgenic mouse ApoE3Leiden with a humanized lipoprotein profile [29]. The aim of our study was to search for processes differentially regulated during high-fat feeding and to identify novel, site-specific functions of adipose tissue. We observed that lipid metabolism and fatty acid pattern in adipose tissue proximal to the reproductive tract differ from fat at other locations and propose that dietary essential fatty acids are accumulated in this area as a result of sex-steroid mediated suppression of lipogenesis, providing a local supply of PUFAs for the epididymis.

## Materials and Methods

### Ethics Statement

All animals received humane care according to the criteria outlined in the “Guide for the Care and Use of Laboratory Animals” prepared by the National Academy of Sciences and published by the National Institutes of Health (NIH). All animal experiments were approved by an independent institutional ethics committee on animal care and experimentation (Dierethische Commissie DEC, Zeist, The Netherlands). Male ApoE3-Leiden transgenic (E3L) mice (n = 100 in total) were from TNO-BioSciences, Gaubius Laboratory, Leiden, The Netherlands.

### Mouse strain, diet and growth conditions

Samples for microarray, histology and lipidomics were obtained from a high-fat feeding experiment previously described [30]. Briefly, male ApoE3Leiden (ApoE3L) transgenic mice [29] were housed in groups (n≤4) with access to water and diet *ad libitum*. The relative humidity in the animal facility was 50–60% and the temperature 21–22°C. A light cycle from 6 am to 6 pm was applied. Prior to high-fat diet (HFD) feeding animals received standard chow (3.3% fat (w/w; 0.6% saturated-, 0.6% monounsaturated (MUFA) and 2.0% PUFA), 19% protein, 36.5% starch and 4.7% sugar) (Sniff R/M H chow, Research Diets, Uden, The Netherlands). Mice were 12 weeks of age and the average weight was 29.6 g (Table S2) at start of the experimental feeding. The HFD contained 24% beef tallow and was composed of 24% fat (w/w; 12% saturated, 10% monounsaturated fatty acids and 1% PUFA), 21% protein and 35% carbohydrates (19% sugars) (diet 4031.05; Hope Farms, Woerden, The Netherlands). The feeding experiment lasted up to 12 weeks, and animals were sacrificed at 0, 1, 6, 9 and 12 weeks. The mice were euthanized at 13.00 after 5–hour fasting. At each time-point mesenteric, inguinal subcutaneous and epididymal adipose depots were harvested. The whole depots were snap-frozen immediately in liquid nitrogen, and stored at −80°C until use.

**Figure 1:**
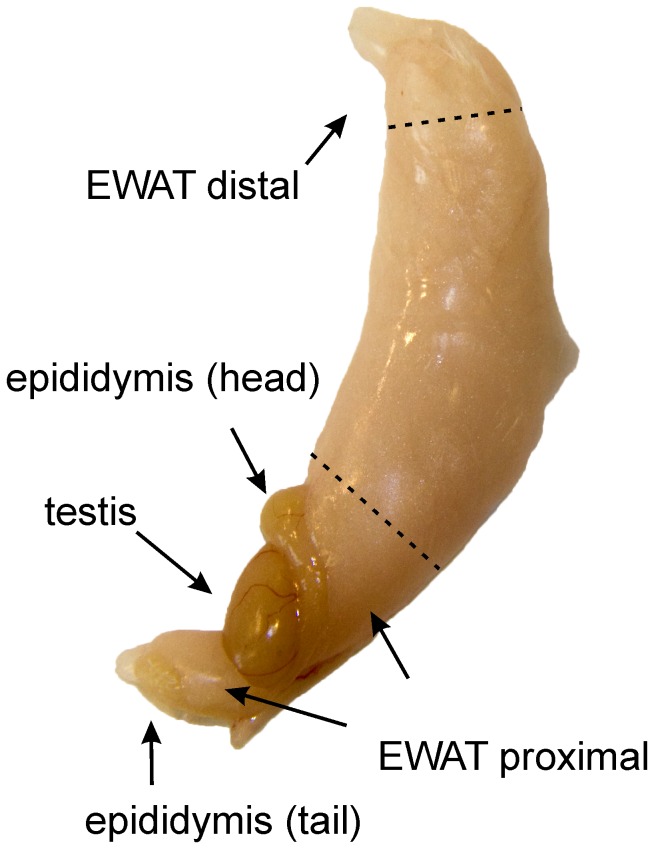
Location of the proximal and distal section of the epididymal adipose tissue (EWAT). Adipose tissue at the distal end of the depot, limited by the upper dashed line, is denoted distal adipose tissue. Adipose tissue associated with the head and the tail of epididymis, indicated by the lower dashed line, is denoted proximal adipose tissue.

Another study was performed for RT-PCR, lipidomics analyses and radioactive tracer experiments. Male ApoE3L mice were housed in groups of 2–3 animals and fed standard chow (4.2% fat (w/w; 0.7% saturated, 1.2% MUFA and 1.6% PUFA), 22.3% protein, 33.9% starch and 5.7% sugars) (Beekay feeds, B & K Universal Ltd., Nittedal, Norway) and water *ad libitum*. Mice were sacrificed at 12–14 weeks of age. The animals were fasted for 5 hours before being euthanized by cervical dislocation at 13.00. Mesenteric and inguinal subcutaneous adipose tissue (whole depots) and epididymal adipose tissue located within 10 mm from the epididymis (proximal) and within 10 mm from the distal end of the epididymal fat depot (distal) were harvested (Figure 1). Fat samples were snap-frozen immediately in liquid nitrogen, and stored at −80°C until analyses, except for some samples immediately used for *ex vivo* analysis of *de novo* lipogenesis and fatty acid accumulation in tissue explants.

The high-fat feeding experiment was a part of the European Nutrigenomics Organisation (NuGO) proof of principle study (PPS) [31].

### Microarray analyses

Total RNA was extracted from adipose tissue using RNAzol (Campro Scientific, Veenendaal, The Netherlands) and glass beads according to the manufacturer's instructions. The integrity of each RNA sample was examined by Agilent Lab-on-a-chip technology using the RNA 6000 Nano LabChip kit and a bioanalyzer 2100 (Agilent Technologies, Amstelveen, the Netherlands) [32]. Affymetrix MOE430-2.0 microarrays were used to determine global gene expression (Affymetrix, Santa Clara, CA). Individual microarrays were performed for each sample. Ten micrograms of RNA were used for one cycle cRNA synthesis (Affymetrix, Santa Clara, CA). Hybridization, washing, and scanning were done according to standard Affymetrix protocols. Array images were processed using packages from the Bioconductor project [33]. Array data have been uploaded to ArrayExpress (http://www.ebi.ac.uk/microarray-as/ae/) (accession number E-TABM-884). All uploaded array data are MIAME compliant.

### Microarray quality control, data filtering and statistical analyses

The number of arrays at time-point 0, 1, 6, 9 and 12 weeks were 4, 5, 7, 3, 6 for epididymal adipose tissue (EWAT); 5, 6, 4, 6, 5 for subcutaneous adipose tissue (SWAT); and 3, 5, 4, 5, 7 for mesenteric adipose tissue (MWAT), respectively.

Quality control was performed by using the Quality Control pipeline on the Madmax microarray analysis server at Wageningen University, the Netherlands (https://madmax.bioinformatics.nl).

Mesenteric and subcutaneous adipose tissue exhibited expression of lymphocyte markers reflecting the abundance of lymph nodes within these depots [14]. For most samples the expression levels of lymphocyte markers were equal. However, three MWAT samples and four SWAT samples, randomly distributed over the different time-points, lacked expression of lymphocyte specific genes indicating that no lymph nodes were present in these sample. To retain within-depot consistency these samples were excluded from the data analyses. Samples containing immuno-specific transcripts were identified by hierarchical clustering. Average linkage clustering was performed by using uncentered correlation [34] (Figure S1 and Table S3).

Normalization was performed in R using the gcRMA algorithm [35].

An IQR (inter quartile range) offset of 0.25 and intensity offset of 20 (log2 4.3) for at least 1 array/gene was applied. This reduced the number of probesets from 15488 to 8849.

For comparison between depots at individual time-points, Tukey post hoc analysis was applied.

Time course analysis of gene expression was performed using EDGE software [36]. Each depot was analyzed separately. Before analysis the data were log2 transformed and a set value of 10 was added to the expression value to avoid random effects at low expression levels. Four degrees of freedom were applied for the analysis. When used this way the EDGE algorithm is identical to one way ANOVA analysis.

Analysis of enrichment of regulated genes within functional categories (gene ontology categories) [37] was performed using the software Metacore (GeneGO, St. Joseph, MI). Genes with an ANOVA p-value <0.05 (4 degrees of freedom) were considered regulated in response to high-fat diet and included in the analysis.

The results of the enrichment calculation were filtered for GO categories that were significantly enriched (p<0.005) in one or two depots but less enriched (p>0.1) in the other depot(s). By using this filter we aimed to identify processes with major differences in regulation between adipose depots.

### Quantitative RT-PCR

Total RNA was reversely transcribed in 20 µL by High Capacity cDNA Reverse Transcription Kit including RNase inhibitor (Applied Biosystems, Foster City, CA) according to manufacturer's protocol. Real-time PCR was performed using TaqMan probes on a 7900HT Fast Real-Time PCR System (Applied Biosystems, Foster City, CA). The following genes were analyzed: Mm00662319_m1 (Fasn); Mm00652520_m1 (Acly); Mm00442688_m1 (Ar). Relative expression was calculated by the ΔΔCt method [38] using Gapdh as endogenous house-keeping gene. The experiment was performed with four biological replicates.

### Lipogenesis and fatty acid accumulation

Immediately after harvest, 10–30 mg adipose tissue samples were cut into small pieces (<1 mm), rinsed in PBS and incubated in 200 µL labeling buffer (Dulbecco's phosphate-buffered saline (DPBS; with Mg2+ and Ca2), 10 mM HEPES (Sigma, St. Louis, MO), 80 µM oleic acid (Nu-Chek Prep, Inc, Elysian, MN) bound to BSA at a ratio of 2.5∶1 and 4 mM glucose) supplemented with either [^14^C]glucose, [^14^C]oleic acid (OA) (Perkin-Elmer, Waltham, MA) or [^14^C]docosahexaenoic acid (DHA) (2.5 µCi/mL)(Hartmann analytic, Braunschweig, Germany). Samples were incubated for 2 h at 37°C. After being rinsed five times in PBS, tissue samples were homogenized with an Ultratorax and lipids were extracted according to Folch *et al.* [39]. To isolate triacylglycerols, solubilized lipids were taken to dryness and the residual extracts were redissolved in 200 µL hexane and separated by thin-layer chromatography (TLC) using hexane-diethyl ether-acetic acid (80/20/1; v/v/v) as developing solvent. The TLC foils were cut into scintillation vials containing Ultima Gold™ scintillation cocktail (Perkin-Elmer, Waltham, MA), and radioactivity was counted in a Wallac 1414 liquid scintillation spectrometer (Perkin-Elmer, Waltham, MA). The experiment was performed with five biological replicates.

### Lipidomics analysis

Extraction and methylation were performed on adipose tissue and spermatozoa (1–2 mg) transferred to 2 mL GC vials. Samples were directly methylated with 3 M methanolic HCL (Supelco33050-U), placed on Thermo mixer at 80°C and mixed at 450 rpm for 2 hours. Thereafter, samples were neutralized with 3M KOH in water and fatty acid methyl esters were extracted in hexane.

GC analysis was performed directly from this solution using a 6890N GC with a split/splitless injector, a 7683B automatic liquid sampler, and flame ionization detection (Agilent Technologies, Palo Alto, CA). Separations were performed with a SP-2380 (30 m ×0.25 mm i.d. ×0.25 µm film thickness) column from Supelco with injection volumes of 0.5 µL.

Statistical analyses of fatty acids were performed for separate as well as for categories of fatty acids. Saturated fatty acids included 14∶0, 15∶0, 16∶0, 17∶0, 18∶0, 20∶0, 22∶0 and 24∶0) whereas MUFA include 16∶1, 18∶1, 20∶1, 22∶1, 24∶1.

### Histology and determination of adipocyte cell size

The samples were fixed overnight by immersion at 4°C in 4% formaldehyde in 0.1 M phosphate buffer at pH 7.4. Then, they were dehydrated, cleared, and paraffin-embedded. Three µm thick sections from two different levels (200 µm apart) were stained with hematoxylin and eosin, examined with a Nikon Eclipse E800 light microscope (Nikon, Japan) using an X 10 lens, and digital images were photographed with a Nikon DXM 1200 camera.

An area of two hundred random unilocular adipocytes from each section was measured using a morphometric program (Lucia IMAGE, Version 4.82, Nikon Instruments, Italy). The value for each experimental group with reference to each depot was calculated as the mean of the values obtained from each animal.

### Statistical analysis

Significance analyses of microarray data were performed by one way ANOVA followed by Tukey post hoc test. Analyses of cell size data, adipose tissue mass data, lipidomics data, RT-PCR data, fatty acid uptake and lipogenesis were performed Kruskall-Wallis analysis followed by Mann–Whitney analysis adjusted by Bonferroni correction. Significance analyses of two groups were performed by Mann–Whitney analyses. Correlations were calculated by Pearson correlation analysis with one-sided significance calculated by Cronbach's Alpha.

## Results

### Adipocytes in EWAT are larger than adipocytes in other adipose depots

**Figure 2:**
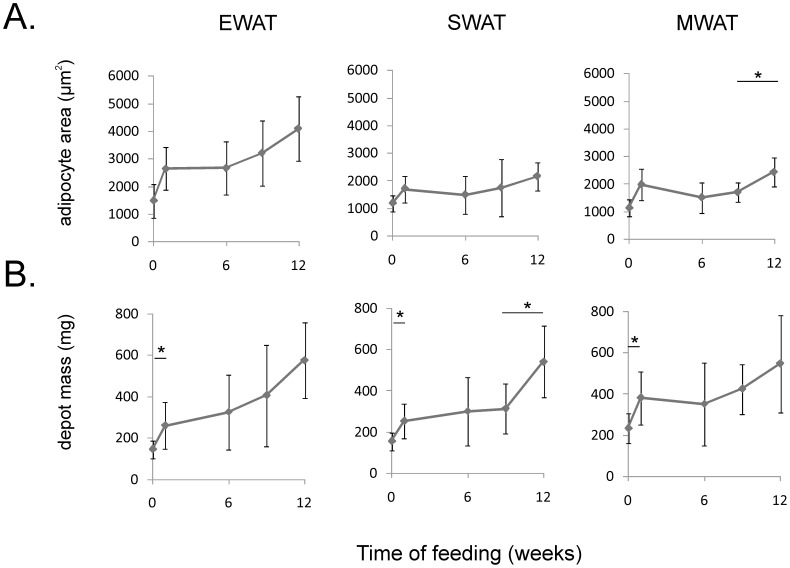
Change of adipocyte cell size and adipose depot mass during high-fat feeding. *A)* Adipocyte cell area in epididymal (EWAT), subcutaneous (SWAT) and mesenteric (MWAT) adipose tissue on high-fat diet (HFD). The areas of adipocytes were determined histologically. The values are based upon 200 measurements from each animal. *B)* Mass of the adipose depots on HFD. 14–15 animals were used for depot weight determination and 6–7 animals for adipocyte size determination. Cell size and depot mass differed over time in all three depots (p<0.05, Kruskall-Wallis analysis, 4 degrees of freedom). *Difference in cell size or depot mass between consecutive time points determined by Mann-Whitney analysis (p<0.05 adjusted to 0.0125 by Bonferroni correction). Error bars indicate standard deviation. Cell size - EWAT: time-point 0–1 weeks U(7) = 6, p = 0.02; 1–6 weeks U(7) = 24, p = 0.94; 6–9 weeks U(7,6) = 16, p = 0.47; 9–12 weeks U(6,7) = 11, p = 0.15; SWAT: time-point 0–1 weeks U(7) = 7, p = 0.02; 1–6 weeks U(7) = 16, p = 0.27; 6–9 weeks U(7,6) = 17, p = 0.56; 9–12 weeks U(6,7) = 9, p = 0.09; MWAT: time-point 0–1 weeks U(7) = 6, p = 0.02; 1–6 weeks U(7,6) = 11, p = 0.15; 6–9 weeks U(6,6) = 10, p = 0.2; 9–12 weeks U(6,7) = 3, p = 0.01; Depot mass - EWAT: time-point 0–1 weeks U(15) = 41, p = 0.003; 1–6 weeks U(15) = 92, p = 0.39; 6–9 weeks U(15,14) = 86.5, p = 0.42; 9–12 weeks U(14,15) = 51, p = 0.02; SWAT: time-point 0–1 weeks U(15) = 26, p = 0.0003; 1–6 weeks U(15) = 108, p = 0.85; 6–9 weeks U(15,14) = 92, p = 0.57; 9–12 weeks U(14,15) = 31, p = 0.001; MWAT: time-point 0–1 weeks U(15) = 29, p = 0.0005; 1–6 weeks U(15,14) = 83, p = 0.33; 6–9 weeks U(14,14) = 60, p = 0.08; 9–12 weeks U(14,15) = 71, p = 0.13.

Adipocyte size was estimated by measuring adipocyte areas histologically. At start of the feeding experiment, EWAT adipocyte areas were slightly larger than adipocyte areas in MWAT and SWAT. After 12 weeks on HFD, EWAT adipocyte areas were approximately twice as large as cell areas in the other depots (Figure 2A). In relative measurements the average cell area increased by 250% and 230% in EWAT and MWAT, respectively, and by 140% in SWAT. Surprisingly, adipocyte area did not increase between week 1 and 9 of HFD. Between week 9 and 12 growth continued, at least in MWAT. The average depot size increased by 300%, 250% and 130% for EWAT, SWAT and MWAT, respectively (Figure 2B).

### Acetyl-CoA biosynthesis is altered by HFD in MWAT and SWAT but not in EWAT

To investigate how gene expression is altered in different adipose tissues, RNA was prepared from epididymal, subcutaneous, and mesenteric fat from mice fed HFD for 0, 1, 6, 9 and 12 weeks, and microarray analyses were conducted. Regulated genes were identified by ANOVA analysis.

Using a p-value threshold of <0.05 and four degrees of freedom, 1658, 1466 and 536 genes were identified as regulated in response to HFD in EWAT, SWAT and MWAT, respectively. Of a total of 3043 regulated genes, only 62 were regulated in all three depots, whereas 2488 were regulated in only one of the depots (1230, 978 and 280 genes in EWAT, SWAT and MWAT, respectively). Thus, our results indicate that major differences in control of gene expression prevail between different adipose tissues.

Gene Ontology (GO) enrichment analysis was performed to identify processes linked to phenotypic differences between adipose depots (Table 1). To identify differentially regulated processes the dataset was filtered for functional categories enriched (p<0.005) in one or two depots while being less enriched (p>0.1) in the other depot(s). By applying dual significance thresholds, processes with major differences in regulation between adipose tissues could be identified. Interestingly, functional categories associated with pyruvate metabolism and acetyl-CoA biosynthesis were highly enriched for regulated genes in SWAT and MWAT but not in EWAT. It was also observed that genes linked to cell cycle progression and development were regulated in SWAT and EWAT, but not in MWAT. However, the expression of many of these genes exhibited enhanced basal expression in MWAT compared to the other depots. Other differentially regulated GO categories included pentose metabolism and sterol metabolism being enriched in regulated genes in MWAT and SWAT, but not in EWAT (Table 1).

**Table 1:** Enrichment of regulated genes within functional categories in adipose depots.

		p-value	
GO category	SWAT	MWAT	EWAT
**Enriched in MWAT and SWAT**			
Acetyl-CoA biosynthesis, metabolism and biosynthesis from pyruvate, pyruvate metabolism	4.1×10^−5^	0.00061	0.54
Coenzyme and cofactor biosynthetic processes	1.8×10^−6^	1.3×10^−5^	0.43
Pentose metabolism and biosynthesis, D-ribose/ ribose phosphate biosynthesis	1.5×10^−5^	0.0033	0.23
Sterol metabolic processese	0.00036	0.0047	0.41
**Enriched in SWAT**			
Quinone cofactor biosynthetic processes	0.22	0.00013	0.58
**Enriched in MWAT**			
Regulation of lipid biosynthetic processes	0.00010	0.22	0.17
**Enriched in SWAT and EWAT**			
Cell division	0.93	0.0039	1.3×10^−5^
M phase/M phase of mitotic cycle	0.99	0.0017	1.3×10^−5^
Mitosis	0.98	0.0031	0.00019
Negative regulation of biological, developmental and multi-cellular organismal processes	0.88	0.0025	0.00018
Regulation of biological quality	0.32	0.0010	7.1×10^−5^
Phosphorus metabolic processes	0.27	0.0027	0.00047

Enrichment of genes regulated during high-fat diet within Gene Ontology (GO) categories were analyzed for mesenteric (MWAT), subcutaneous (SWAT) and epididymal (EWAT) adipose tissue. To identify processes with major differences in enrichment of regulated genes between depots, the results were filtered for GO categories significantly enriched (p<0.005) in one or two depots but less enriched (p>0.1) in the other depot(s). Gene regulation in response to high-fat diet was determined by ANOVA (p-value <0.05, 4 degrees of freedom, N at time-point 0, 1, 6, 9 and 12 weeks were 4, 5, 7, 3, 6 for EWAT; 5, 6, 4, 6, 5 for SWAT; and 3, 5, 4, 5, 7 for MWAT, respectively). GO category enrichment analysis was performed in Metacore.

Taken together, gene regulation in response to HFD, especially for genes encoding enzymes involved in acetyl-CoA synthesis, differs markedly between adipose depots.

### Reduced *de novo* lipogenesis in SWAT and MWAT, and enhanced fatty acid desaturation in EWAT

**Figure 3:**
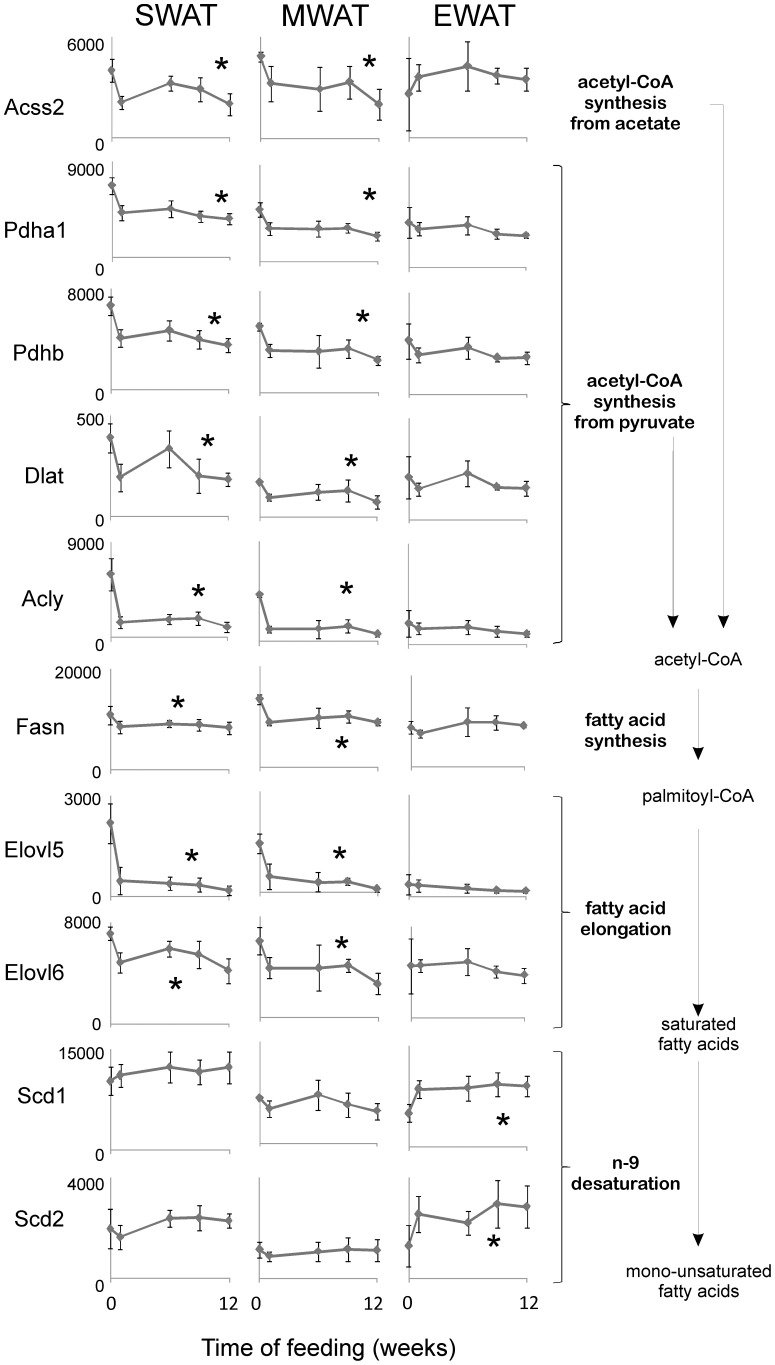
Expression of genes encoding enzymes in fatty acid synthesis and processing during basal conditions (chow) and high-fat feeding. Expression of acetyl-CoA synthetase short-chain family member 2 (Acss2), pyruvate dehydrogenase α1 (Pdha1), pyruvate dehydrogenase β (Pdhb) dihydrolipoamide S-acetyltransferase (Dlat), ATP citrate lyase (Acly), fatty acid synthase (Fasn), long chain elongation enzymes Elovl5 and Elovl6 and stearoyl-CoA desaturase 1 and 2 (Scd1/2) in subcutaneous (SWAT), mesenteric (MWAT) and epididymal (EWAT) adipose tissue. The y-axis scale is the same for all three depots. Gene expression is given as relative expression. N at time-point 0, 1, 6, 9 and 12 weeks were 4, 5, 7, 3, 6 for epididymal adipose tissue (EWAT); 5, 6, 4, 6, 5 for subcutaneous adipose tissue (SWAT); and 3, 5, 4, 5, 7 for mesenteric adipose tissue (MWAT), respectively. Error bars indicate standard deviation. Statistically significant changes in expression over time are indicated by *(p<0.05 (one way ANOVA)). SWAT: Acss2 F(4,21) = 10.1, p = 9.8×10^−5^; Pdha1 F(4,21) = 17.9, p = 1.5×10^−6^; Pdhb F(4,21) = 15.3, p = 5.3×10^−6^; Dlat F(4,21) = 8.8, p = 0.0002; Acly F(4,21) = 30.0, p = 2.0×10^−8^; Fasn F(4,21) = 8.8, p = 0.0002; Elovl5 F(4,21) = 2.9, p = 0.04; Elovl6 F(4,21) = 27.5, p = 4.4×10^−8^; Scd1 F(4,21) = 0.9, p = 0.47; Scd2 F(4,21) = 2.2, p = 0.10; MWAT: Acss2 F(4,18) = 4.3, p = 0.013; Pdha1 F(4,18) = 9.9, p = 0.0002; Pdhb F(4,18) = 7.2, p = 0.001; Dlat F(4,18) = 4.6, p = 0.009; Acly F(4,18) = 29.9, p = 9.9×10^−8^; Fasn F(4,18) = 5.3, p = 0.0.005; Elovl5 F(4,18) = 9.7, p = 0.0002; Elovl6 F(4,18) = 14.4, p = 1.9×10^−5^; Scd1 F(4,18) = 2.0, p = 0.14; Scd2 F(4,18) = 0.42, p = 0.79; EWAT: Acss2 F(4,20) = 1.1, p = 0.37; Pdha1 F(4,20) = 1.9, p = 0.19; Pdhb F(4,20) = 2.3, p = 0.09; Dlat F(4,20) = 2.0, p = 0.12; Acly F(4,20) = 1.4, p = 0.25; Fasn F(4,20) = 0.82, p = 0.53; Elovl5 F(4,20) = 1.7, p = 0.28; Elovl6 F(4,20) = 1.2, p = 0.32; Scd1 F(4,20) = 5.1, p = 0.005; Scd2 F(4,20) = 3.4, p = 0.027.

Pathways involved in acetyl-CoA synthesis and pyruvate metabolism exhibited several regulated genes in MWAT and SWAT but not in EWAT. Acetyl-CoA is a precursor of *de novo* lipogenesis. To investigate in detail how fatty acid synthesis and processing are affected in adipose tissues by HFD, expression levels of genes involved in acetyl-CoA production and the down-stream metabolic pathways of fatty acid synthesis, elongation and desaturation, were plotted against time (Figure 3).

All eight regulated genes involved in acetyl-CoA synthesis, fatty acid synthesis and elongation exhibited similar expression patterns. In MWAT and SWAT gene expression was reduced. ANOVA analysis followed by post hoc analysis showed that changes in gene expression mainly occurred during the first week of HFD (data not shown). Expression of the genes encoding ATP citrate lyase and Elovl6 were reduced by approximately 85%, whereas expression of other genes was reduced by 25–60%. In EWAT none of these genes were significantly regulated. On chow diet gene expression in EWAT was markedly lower than in MWAT and SWAT.

Control of delta-9 desaturases, stearoyl-CoA desaturase 1 and 2 (Scd1/2) catalyzing the conversion of saturated fatty acids to MUFA, differed from the control of genes involved in fatty acid synthesis and elongation. Both Scd1 and Scd2 were approximately 2-fold enhanced in EWAT during the first week of HFD, whereas the expression level in MWAT and SWAT remained unaltered.

These results show that *de novo* lipogenesis is stably low in EWAT during all the feeding period, whereas HFD reduced *de novo* lipogenesis in SWAT and MWAT and enhanced delta-9 desaturase activity in EWAT. Apparently, fatty acid synthesis and desaturation are not co-regulated under our experimental conditions.

### Omega-6 and omega-3 fatty acids in EWAT are replaced by MUFA during HFD

The HFD used in our present study contained about 12% (w/w) saturated fatty acids (SFA), 10% MUFA and 1% PUFA and induced the expression of delta-9 desaturase in EWAT. To investigate how the fatty acid composition of EWAT is affected by HFD, targeted lipidomics was performed at time-point 0 and 12 weeks.

**Figure 4:**
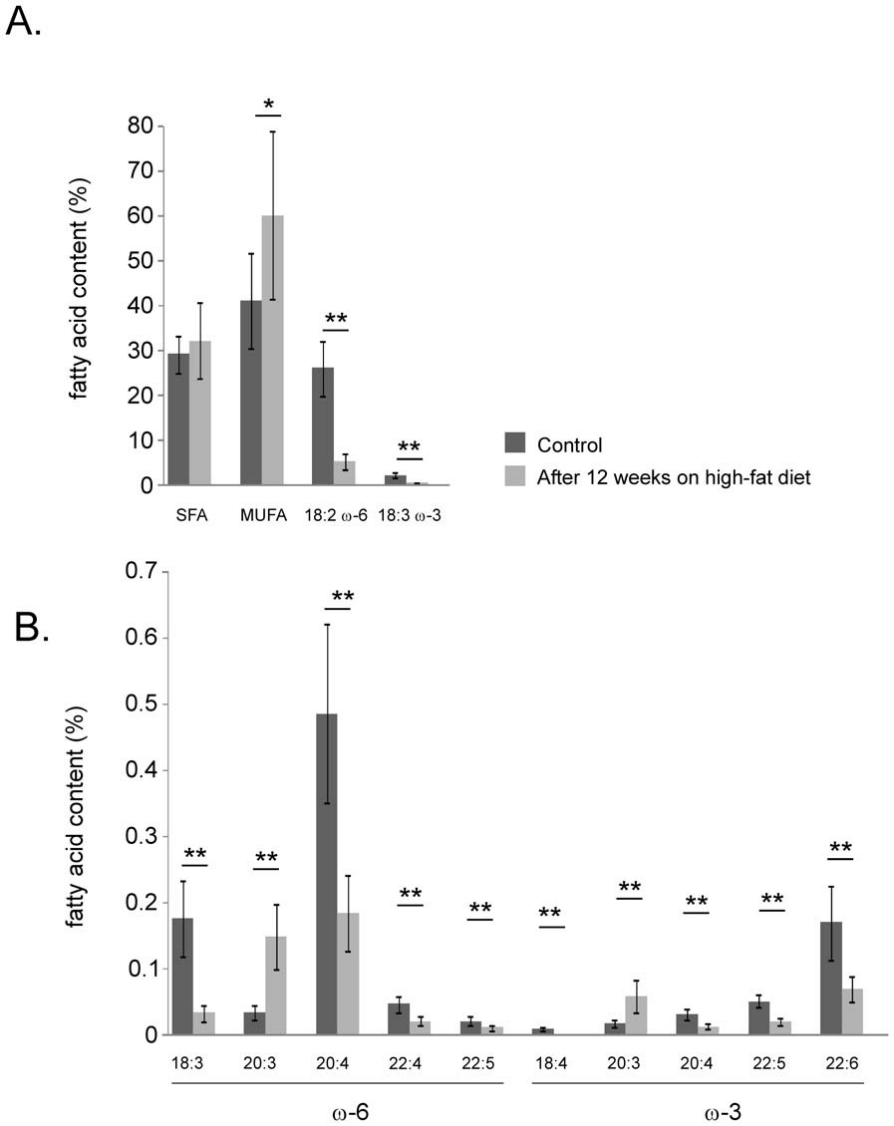
Lipidomics analyses of epididymal adipose tissue during basal conditions (chow) and high-fat feeding. Proportions of total fatty acids in epididymal adipose tissue at 0 and 12 weeks on high-fat diet: *A)* Saturated fatty acids (SFA) monounsaturated fatty acids (MUFA), linoleic acid (18∶2 ω-6) and α-linolenic acid (18∶3 ω-3). *B)* Low abundance omega-6 (ω-6) and omega-3 (ω-3) fatty acids. Error bars indicate standard deviation. *p<0.05 (Mann–Whitney analysis). SFA U(8) = 23, p = 0.34; MUFA U(8) = 14, p = 0.06; 18∶2 ω-6 U(8) = 0, p = 0.0007; 18∶3 ω-3 U(8) = 0, p = 0.0007; 18∶3 ω-6 U(8) = 0, p = 0.0007; 20∶3 ω-6 U(8) = 0, p = 0.0007; 20∶4 ω-6 U(8) = 0, p = 0.0007; 22∶4 ω-6 U(8) = 0, p = 0.0007; 22∶5 ω-6 U(8) = 3, p = 0.0023; 18∶4 ω-3 U(8) = 0, p = 0.0007; 20∶3 ω-3 U(8) = 0, p = 0.0007; 20∶4 ω-3 U(8) = 0, p = 0.0007; 22∶5 ω-3 U(8) = 0, p = 0.0007; 22∶6 ω-3 U(8) = 0, p = 0.0007.

The proportion of SFA in adipose tissue did not change during high-fat feeding but remained constant at approximately 30% (Figure 4A). However, the fractions of omega-6 and omega-3 fatty acids were dramatically reduced. The most abundant PUFAs, linoleic acid (LA) and α-linolenic acid (Figure 3A), decreased from 26% to 5% and from 2% to 0.3%, respectively, while other species in the ω-3 and ω-6 PUFA series, such as arachidonic acid (20∶4 ω-6) and docosahexaenoic acid (22∶6 ω-3), were reduced by approximately 60% (Figure 3B). The fatty acids dihomo-gamma-linolenic acid (20∶3 ω-6) and eicosatrienoic acid (20∶3 ω-3) posed exceptions from the trend and were both enriched on HFD. Strikingly, PUFAs were partly replaced by oleic acid (18∶1), which increased from 41 to 60%.

The present data do not allow an estimation of how much the increased delta-9 desaturase expression in response to HFD affects the proportions of fatty acids stored in EWAT, but we noticed that the MUFA fraction was markedly increased whereas the fraction containing saturated fatty acids remained relatively stable during the experiment.

### Fatty acid synthesis and accumulation differs in different parts of EWAT

**Figure 5:**
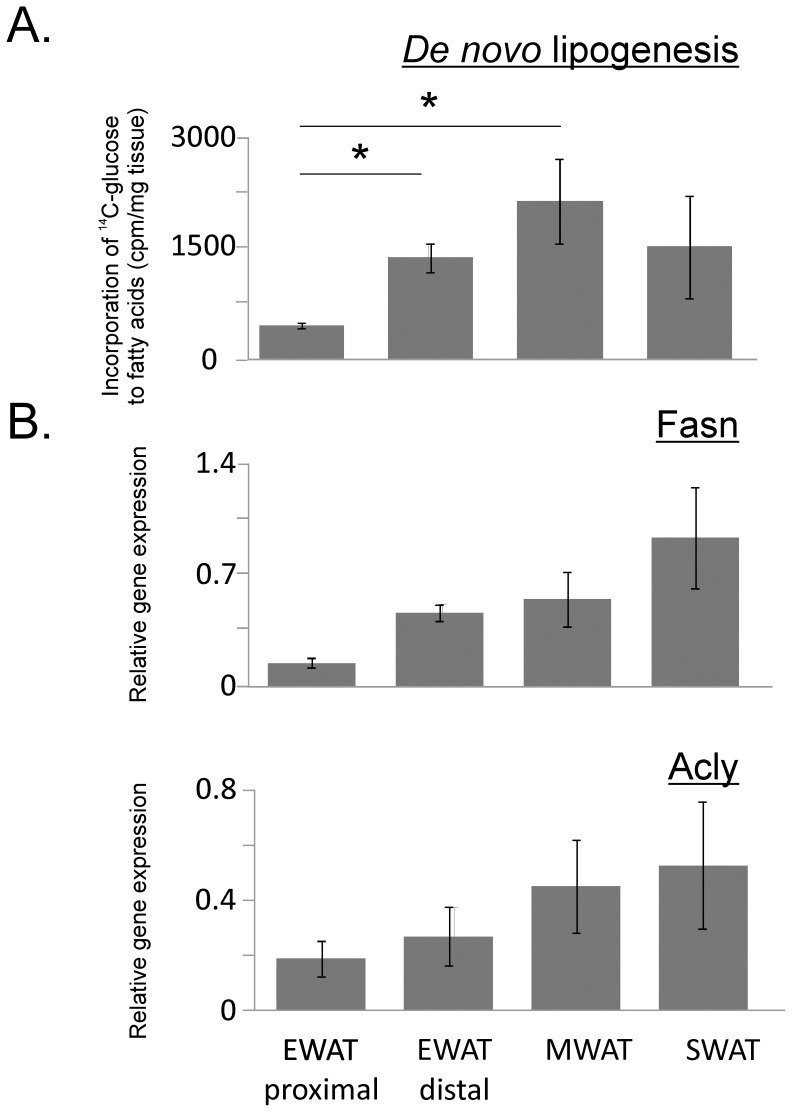
Lipogenesis and expression of genes involved in lipogenesis in proximal epididymal adipose tissues compared to other adipose tissues during chow feeding. *A) De novo* lipogenesis determined by incorporation of [^14^C] from radiolabeled glucose in triacylglycerols in explants from the proximal and distal zone of epididymal (EWAT), subcutaneous (SWAT) and mesenteric (MWAT) adipose tissue. *B)* Expression of genes encoding fatty acid synthase (Fasn) and ATP citrate lyase (Acly) determined by RT-PCR. *De novo* lipogenesis and Fasn expression but not Acly expression differed between depots (p<0.05, Kruskall-Wallis analysis, 3 degrees of freedom). *Difference between proximal EWAT and other adipose tissues determined by Mann-Whitney analysis (p<0.05 adjusted to 0.017 by Bonferroni correction). Experiments were performed with 4–5 animals. Error bars indicate standard deviation. *de novo* lipogenesis: MWAT-EWAT proximal U(4,5) = 0, p = 0.014; SWAT-EWAT proximal U(5) = 5, p = 0.12; EWAT proximal-EWAT distal U(5) = 0, p = 0.009; Fasn: MWAT-EWAT proximal U(4) = 2, p = 0.08; SWAT-EWAT proximal U(4) = 0, 0.02; EWAT proximal-EWAT distal U(4) = 0, p = 0.02; Acly: MWAT-EWAT proximal U(4) = 2, p = 0.08; SWAT-EWAT proximal U(4) = 2, 0.08; EWAT proximal-EWAT distal U(4,3) = 5, p = 0.72.

Regulation of fatty acid metabolism differed between EWAT and the other adipose depots. We also observed that two EWAT samples at baseline exhibited high expression of *de novo* lipogenesis genes, whereas the expression levels for the two other samples were much lower (data not shown). To investigate if the observed variation is caused by regional differences within the EWAT depot, samples from the proximal and distal part of EWAT were analyzed. The proximal and distal regions comprised adipose tissue within 10 mm from epididymis and within 10 mm from the distal end of the EWAT depot, respectively (Figure 1). MWAT and SWAT samples were also included in the analyses. *de novo* lipogenesis was determined by measuring the transfer of [^14^C] from glucose to lipids in fresh adipose tissue explants. Strikingly, *de novo* lipogenesis in proximal EWAT was 70% lower compared to distal EWAT and SWAT, and 80% lower as compared to MWAT (Figure 5A). The low *de novo* lipogenesis activity in especially proximal EWAT was confirmed by gene expression analysis of fatty acid synthetase (Fasn) and ATP citrate lyase (Acly) (Figure 5B).

**Figure 6:**
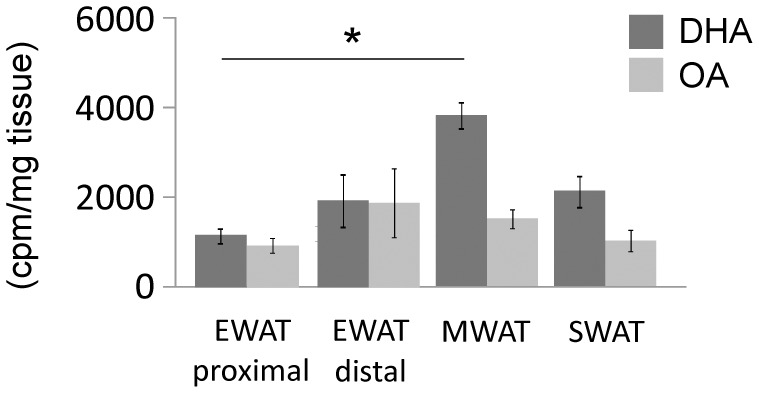
Accumulation of triacylglycerol fatty acids in adipose tissue explants. Cell-associated incorporation of [^14^C]radiolabeled docosahexaenoic acid (DHA) and oleic acid (OA) in cellular triacylglycerols in the proximal and distal zone of epididymal (EWAT), subcutaneous (SWAT) and mesenteric (MWAT) adipose tissues. Adipose tissue explants from animals on chow diet were incubated in medium containing fatty acids bound to BSA, lipids were extracted and triacylglycerols were isolated by thin layer chromatography. The experiments were performed with 4–5 animals. DHA uptake but not OA uptake differed between depots (p<0.05, Kruskall-Wallis analysis, 3 degrees of freedom). *Difference determined by Mann-Whitney analysis (p<0.05 adjusted to 0.017 by Bonferroni correction). Error bars indicate standard deviation. DHA uptake: MWAT-EWAT proximal U(4,5) = 0, p = 0.014; SWAT-EWAT proximal U(5) = 3, 0.05; EWAT proximal-EWAT distal U(5,4) = 5, p = 0.22; OA uptake: MWAT-EWAT proximal U(4,5) = 3, p = 0.08; SWAT-EWAT proximal U(4,5) = 9, 0.80; EWAT proximal-EWAT distal U(5) = 9, p = 0.46.

To study if accumulation of supplemented fatty acids also differs between proximal EWAT and other depots, fresh adipose tissue explants were incubated in medium containing either radiolabeled oleic acid or radiolabeled docosahexaenoic acid (DHA). Accumulation of the labeled fatty acids was determined by measuring radioactivity in triacylglycerols separated by TLC. Proximal EWAT showed reduced accumulation of fatty acids compared to distal EWAT, MWAT and SWAT (Figure 6).

By dividing the epididymal adipose tissue into a proximal and a distal section we revealed functional zonation in the depot, whereby proximal EWAT exhibited reduced synthesis and accumulation of fatty acid as compared to the two other adipose locations.

### Adipose tissue *de novo* lipogenesis is negatively correlated with expression of the androgen receptor (Ar) on chow diet

Nutrient intake as well as sex-steroids may regulate *de novo* lipogenesis in adipose tissue [40]–[42]. To identify mechanisms that determine site-specific differences for *de novo* lipogenesis, the array dataset was screened for genes encoding members of regulatory pathways and hormone receptors.

**Figure 7:**
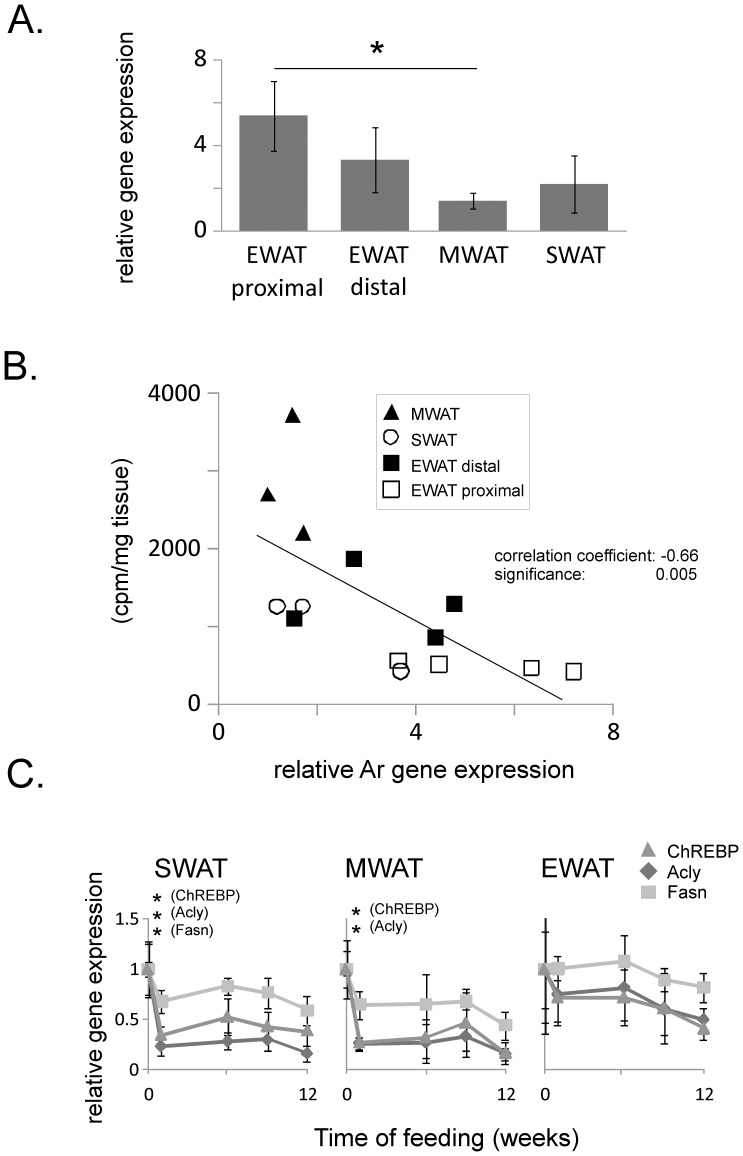
Rate of lipogenesis and expression of putative regulatory factors. *A)* Gene expression levels of the androgen receptor (Ar) in proximal and distal epididymal (EWAT) adipose tissue, in mesenteric (MWAT), and in subcutaneous (SWAT) adipose tissue determined by RT-PCR. *B)* Expression of the gene encoding the androgen receptor (Ar) determined by RT-PCR plotted against rate of lipogenesis measured by incorporation of [^14^C]glucose in TAG in adipose tissue explants. *C)* Expression of carbohydrate responsive element binding protein (ChREBP), ATP citrate lyase (Acly) and fatty acid synthase (Fasn) on 12 weeks of high-fat diet determined by microarray analysis. The expression level at baseline was set to 1 for all genes. (A) Ar expression differed between depots (p<0.05, Kruskall-Wallis analysis, 3 degrees of freedom), *Difference between proximal EWAT and other adipose tissues determined by Mann-Whitney analysis (p<0.05 adjusted to 0.017 by Bonferroni correction). MWAT-EWAT proximal U(5) = 0, p = 0.009; SWAT-EWAT proximal U(5) = 1, 0.02; EWAT proximal-EWAT distal U(5) = 3, p = 0.05 (B) the relation between lipogenesis and Ar expression was determined by Pearson correlation coefficient with one-sided significance calculated by Cronbach's Alpha, (C) *p<0.05 (one way ANOVA with Tukey post hoc analysis, 4 degrees of freedom. Error bars indicate standard deviation. SWAT: ChREBP F(4,21) = 10, p = 0.0001; Acly F(4,21) = 30.0, p = 2.0×10^−8^; Fasn F(4,21) = 8.8, p = 0.0002; MWAT: ChREBP F(4,18) = 8.6, p = 0.0004; Acly F(4,18) = 29.9, p = 9.9×10^−8^; Fasn F(4,18) = 5.3, p = 0.0.005; EWAT: ChREBP F(4,20) = 2.8, p = 0.055; Acly F(4,20) = 1.4, p = 0.25; Fasn F(4,20) = 0.82, p = 0.53.

Testosterone has previously been shown to repress expression of genes encoding lipogenic enzymes in adipose tissue [42]. Array data from our present study showed that expression of the Ar is higher in EWAT than in MWAT and SWAT (data not shown). In a follow-up RT-PCR analysis the increased expression of Ar in EWAT compared to other adipose depots was confirmed (Figure 7A). Furthermore, Ar expression appeared to be higher in proximal EWAT than in distal EWAT, and we observed that Ar expression and *de novo* lipogenesis activity were negatively correlated (Figure 7B). A negative correlation was also observed between expression of Ar and expression of genes encoding fatty acid synthetase (Pearson correlation -0.43, one-sided significance 0.05) and ATP citrate lyase (Pearson correlation −0.43, one-sided significance 0.05). Ar expression was not affected by diet. The negative correlation between Ar expression and *de novo* lipogenesis activity was only observed on chow diet, whereas we did not examine the Ar expression in detail on HFD.

### Expression of *de novo* lipogenesis genes is co-regulated by the transcription factor carbohydrate responsive element binding protein (ChREBP) on HFD

We observed that expression of genes engaged in *de novo* lipogenesis in SWAT and MWAT was strongly regulated on HFD (Figure 3). Little is known about the mechanisms involved in the regulation of *de novo* lipogenesis in adipose tissue in response to diet but the hepatic transcription factors sterol regulatory element binding protein 1c (SREBP1c) and ChREBP have been proposed as nutrient sensors for *de novo* lipogenesis [43]–[45]. Interestingly, we found that ChREBP and genes encoding *de novo* lipogenesis enzymes were tightly co-regulated in SWAT and MWAT (Figure 7C), whereas the expression level of SREBP1c was unaffected by diet.

### Linoleic acid and α-linolenic acid are enriched in EWAT

**Figure 8:**
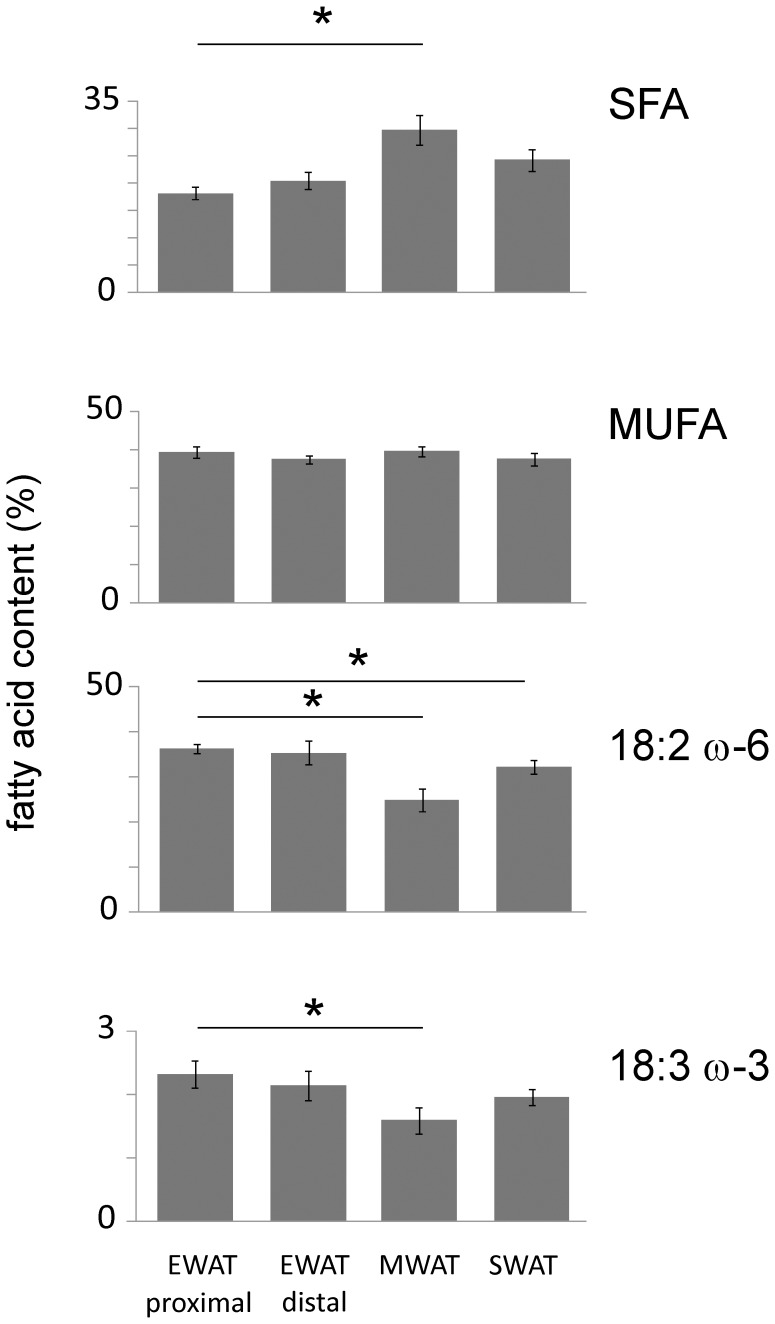
Fatty acid profiles of adipose tissues. Proportions of saturated fatty acids (SFA), monounsaturated fatty acids (MUFA), linoleic acid (18∶2 ω-6) and α-linolenic acid (18∶3 ω-3) in proximal epididymal and distal epididymal (EWAT) adipose tissue, in mesenteric (MWAT), and in subcutaneous (SWAT) adipose tissue on chow feeding. SFA, linoleic acid and α-linolenic acid but not MUFA differed between depots (p<0.05, Kruskall-Wallis analysis, 3 degrees of freedom). *Difference between proximal EWAT and other adipose tissues determined by Mann-Whitney analysis (p<0.05 adjusted to 0.017 by Bonferroni correction). Error bars indicate standard deviation. SFA: MWAT-EWAT proximal U(5) = 0, p = 0.009; SWAT-EWAT proximal U(5) = 1, 0.02; EWAT proximal-EWAT distal U(5) = 12, p = 0.9; MUFA: MWAT-EWAT proximal U(5) = 7, p = 0.25; SWAT-EWAT proximal U(5) = 9, 0.46; EWAT proximal-EWAT distal U(5) = 11, p = 0.75; linoleic acid: MWAT-EWAT proximal U(5) = 0, p = 0.009; SWAT-EWAT proximal U(5) = 0, 0.009; EWAT proximal-EWAT distal U(5) = 10, p = 0.6; α-linolenic acid: MWAT-EWAT proximal U(5) = 0, p = 0.009; SWAT-EWAT proximal U(5) = 2, 0.03; EWAT proximal-EWAT distal U(5) = 7, p = 0.25.

High lipogenic activity in adipose tissue has been reported to be positively correlated with the content of saturated fatty acids [46]. The mechanistic background for this relationship is unknown but saturated fatty acids are the end products of *de novo* lipogenesis and it is possible that local lipogenic rate affects the stoichiometric relationship between stored fatty acids in favor of saturated fatty acids. To investigate if the observed site-specific differences in *de novo* lipogenesis (Figure 5) are related to differences in fatty acid composition, targeted lipidomics was performed on MWAT, SWAT, and the proximal and distal EWAT on chow diet. Indeed, a strong correlation between *de novo* lipogenesis activity and fatty acid composition was found. The total content of saturated fatty acids ranged from 30% in MWAT to 21% in proximal EWAT (Figure 8). Saturated fatty acids with even carbon number (14∶0–20∶0) exhibited similar proportions between depots (Figure 8), whereas the low content of saturated fatty acids with odd carbon numbers (15∶0 and 17∶0) was similar in all depots (data not shown). EWAT was enriched in linoleic acid (18∶2 ω-6) and α-linolenic acid (18∶3 ω-3).

The content of linoleic acid in proximal and distal EWAT was 37% and 36%, respectively, whereas the content in MWAT was 25% and that in SWAT was 32% (Figure 8). The content of α-linolenic acid ranged from 2.3% in proximal EWAT to 1.6% in MWAT. The amount of MUFA and of most PUFA was similar in all four depots. Besides linoleic acid and α-linolenic acid, eicosapentaenoic acid (EPA, 20∶5 ω-3) was slightly enriched in proximal EWAT, whereas the rare fatty acid eicosenoic acid (20∶1 ω-9) was reduced in EWAT (data not shown).

We conclude that during basal conditions fatty acid composition in adipose depots differs mainly in the content of saturated fatty acids, linoleic acid and α-linolenic acid. EWAT, especially the proximal part, is enriched in linoleic and α-linolenic acid.

## Discussion

We performed a time-resolved, comparative microarray analysis on MWAT, SWAT and EWAT during development of HFD-induced obesity in ApoE3 Leiden mice. Major differences in control of gene expression between depots were recognized emphasizing that adipose depots harvested from a single location is unique and cannot be considered representative for adipose tissue in the whole body.

To identify unique features of individual depots, the dataset was filtered for processes with marked differences in enriched and differentially regulated genes. EWAT differed from the two other examined adipose tissues by its lack of *de novo* lipogenesis control in response to HFD. We demonstrated that *de novo* lipogenesis in EWAT, or more specifically in the proximal zone of EWAT, was lower than in the two other depots on a chow diet and prior to HFD feeding. This depot-specific difference in rate of lipogenesis represents an original finding, as other investigators have previously reported equal expression levels of *de novo* lipogenesis genes in EWAT and SWAT [42]. However, it should be emphasized that only the proximal end of EWAT exhibits low *de novo* lipogenesis and that it is critical how accurately tissue samples are harvested, a fact which is frequently underestimated. The novel observation that EWAT is divided into zones with potentially different physiological properties highlights that adipose tissues are diverse and that the traditional division of adipose tissues based on anatomical features may be inadequate in terms of functional properties.

We also observed that *de novo* lipogenesis rate and the content of even numbered saturated fatty acids were positively correlated. Many of these fatty acids are produced during *de novo* lipogenesis and the observed correlation is probably linked to differences in local fatty acid synthetic rates. *de novo* lipogenesis activity and the concentration of saturated fatty acids have previously been shown to be positively correlated in human adipocytes [46]. The proportions of linoleic acid and α-linolenic acid were increased in EWAT compared to other adipose depots. The accumulation of these dietary fatty acids may be caused by the low lipogenic activity in EWAT.

It was reported recently that the lipogenic rate in adipose tissue is increased after castration [42]. The increase was observed both in EWAT and SWAT and was specific for male mice indicating that testosterone might exert an inhibitory effect on *de novo* lipogenesis. We found that expression of the Ar is up to 10-fold higher in EWAT than in MWAT and SWAT, and that *de novo* lipogenesis and the expression of Ar is negatively correlated. Depot-specific differences in response to testosterone pose an attractive mechanistic background for the observed differences in lipogenic activity during basal conditions. The putative role of sex-steroids in the control of *de novo* lipogenesis indicates a cross-talk between reproductive organs and the local fat pad, where testosterone secretion from testis causes local accumulation of essential fatty acids. This hypothesis is supported by the observation that testosterone injected into testis and epididymis is circulated to the epididymal fat pad [47]. A similar relationship between lymph nodes and adipose tissue has been described by Pond and coworkers [14], [48] demonstrating that adipocytes associated with lymphoid structures accumulate precursors for eicosanoids and docosanoids, and release them when lymphocytes are activated in response to immune stimuli. Even though Ar expression and *de novo* lipogenesis exhibit a strong positive relation we do not present any mechanistic proof for the suggested sex-steroid mediated control of *de novo* lipogenesis and other factors, e.g. the difference in temperature between the testicles and central parts of the body, might be of importance for fatty acid accumulation. It should also be emphasized that most animals, including humans, have small epididymal adipose depots. It is possible that local metabolic variations in epididymal adipose tissue are absent in humans and that the whole depot should be considered proximal to epididymis.

Carbohydrate response-element binding protein (ChREBP), along with SREBP1c and LXRα, may be involved in dietary regulation of hepatic *de novo* lipogenesis [44], [49], [50]. ChREBP is activated by glucose and inhibited by glucagon and fatty acids [45], [51], [52]. ChREBP is present in human as well as rodent adipose tissue (7, 21–24) but an active role of ChREBP in the control of adipose tissue *de novo* lipogenesis has not been demonstrated. Cell culture experiments have shown that ChREBP expression is induced by troglitazone, glucose and insulin, and reduced in response to fatty acids [53], whereas previous *in vivo* studies have suggested that ChREBP is unresponsive to HFD and fasting but enhanced in response to re-feeding [40], [53]. Our data show that adipose tissue ChREBP is markedly reduced in response to HFD in a depot-specific manner and that the expression profile of ChREBP is closely correlated to the expression of *de novo* lipogenesis genes. ChREBP activation appears to be mainly post-transcriptionally regulated in the liver [54] and additional experiments are required to establish if ChREBP is involved in the regulation of adipose tissue *de novo* lipogenesis.

**Figure 9:**
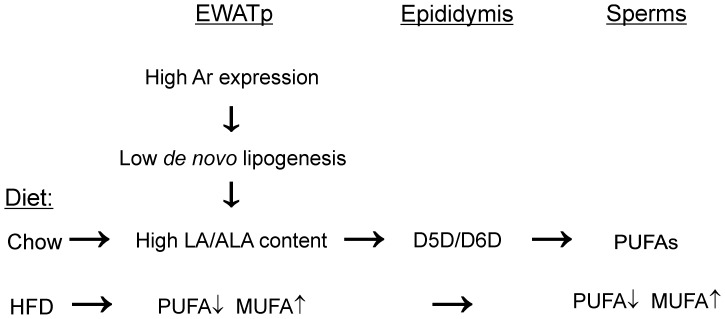
Model for site-specific androgen control of lipogenesis promoting enrichment of essential fatty acids in the proximal epididymal adipose tissue. Androgen suppression of adipose tissue *de novo* lipogenesis is regulated by androgen receptor (Ar). Ar is highly expressed in proximal epididymal adipose tissue (EWATp). Low rate of *de novo* lipogenesis causes high content of dietary fatty acids and on chow diet the essential fatty acids linoleic acid and α-linolenic acid accumulate. Delta-6 and delta-5 desaturase (D6D and D5D) in epididymis convert linoleic acid and α-linolenic acid into polyunsaturated fatty acids (PUFA) with 20 and 22 carbon atoms required for spermatogenesis. When animals are fed high-fat diet (HFD) rich in saturated fatty acids adipose tissue is depleted of PUFA, potentially resulting in impaired sperm quality and decreased fertility.

A major function of adipose tissue is storage of surplus energy. To carry out this task adipocyte metabolism is flexible and tightly influenced by energy balance. Excess dietary carbohydrates are transformed to fatty acids by *de novo* lipogenesis and stored as triacylglycerols. Thus, it is intriguing that the control of *de novo* lipogenesis in proximal EWAT is suppressed independent of diet, and that nutrient control appears to be abolished by sex-steroid control. We find that sperms are highly enriched in PUFA, especially DHA and arachidonic acid (Table S1) and propose a model where sex-steroids suppress *de novo* lipogenesis in adipose tissue associated with the testicles and thereby optimize this tissue for storage of dietary fatty acids as precursors during spermatogenesis (Figure 9). According to this model linoleic acid and α-linolenic acid are transported from EWAT to epididymis and potentially to testis where they are processed by delta-5 and delta-6 desaturases [55], [56]. Long term diet enriched in saturated and monounsaturated fatty acids will deplete the storage of linoleic acid and α-linolenic acid, and eventually also change the fatty acid composition of the sperms. It has been shown that sperms isolated from animals kept on fat-free diet for six weeks exhibit only slightly reduced levels of PUFA, indicating that stored fatty acids are used as building blocks during spermatogenesis and can sustain the demand over long periods of time with insufficient dietary fatty acid supply [55]. Removal of epididymal adipose tissue abolish spermatogenesis [57], [58] but does not influence Leidig cell structure or testosterone production [58]. Furthermore, a transgenic mouse model lacking white adipose tissue (male A-ZIP/F-1), exhibits reduced fertility independent of leptin and testosterone levels [59], suggesting a potential role for stored fatty acids in the production of viable sperms.

Interestingly, the mobilization of fatty acids from adipose tissue may be selective and depends mainly on fatty acid chain length and the degree of unsaturation [29]. Generally, fatty acids with shorter and more unsaturated carbon chains are more readily mobilized during fat store depletion [34].

We observed that omega-6 and omega-3 fatty acids in EWAT are partly replaced by MUFA during HFD and conclude that the diet used in our study cannot maintain adipose tissue fatty acid composition during 12 weeks diet intervention. This suggests that long term HFD will deplete the reservoir of precursors for AA and DHA stored in adipose tissue, and may impair sperm quality in the long run. It has been shown that obese men exhibit impaired spermatogenesis with reduced sperm count [60] although the role of adipose tissue fatty acid composition in relation to fertility has not been investigated.

Even though we present evidence that PUFA are accumulated proximal to the epididymis and that accumulation may be facilitated by signals produced by the reproductive organs many features of this putative paracrine interaction remains to be investigated. For example, it is still unknown if adipose tissue lipolysis and subsequent recruitment of fatty acids to epididymis and testis can be induced by local signaling. It is also not known whether the observed site-specific differences in fatty acid composition are dependent solely on adipocytes or if other cell types present in adipose tissue, e.g. lymphocytes, may contribute.

Analytically the most challenging aspect of the array study was to cope with depot-specific differences in cell type composition. Approximately 90% of the SWAT and MWAT samples included high levels of lymphocyte markers, and to obtain within-depot uniformity, SWAT and MWAT samples with no expression of lymphocyte markers were excluded. In subsequent analyses of gene expression the excluded samples were used as *ad hoc* controls to identify cell-type dependent differences. Moreover, several genes more expressed in lymphocytes than in adipocytes, were identified by hierarchical clustering of array data, and the genes were excluded from the data analyses (Figure S1, Table S3).

In summary, our results show that the physiological response to HFD differs between adipose depots. EWAT differs from the other depots with respect to control of *de novo* lipogenesis. We find that *de novo* lipogenesis is stably low in the proximal zone of EWAT, and we present a model where storage of PUFA in EWAT is favored by testosterone-mediated inhibition of *de novo* lipogenesis. Furthermore, we suggest that local accumulation of linoleic acid and α-linolenic acid may be an adaptive strategy to provide precursors for epididymal PUFA synthesis.

## Supporting Information

Figure S1 Hierarchical clustering reveals lymphocyte specific genes in adipose tissue samples. Lymphocyte specific genes are present in some adipose tissue samples but not in others. A cluster with a correlation coefficient of 0.97 containing lymphocyte specific genes was identified by hierarchical clustering of array data from mesenteric (MWAT), subcutaneous (SWAT) and epididymal (EWAT) adipose tissue samples (dendrogram and heat-map representing the whole data-set shown to the left, blow-up of cluster containing lymphocyte specific genes shown to the right). Within this cluster genes are expressed in 28 of 32 SWAT samples, 23 of 26 MWAT samples and 0 of 25 EWAT samples. The MWAT and SWAT arrays not expressing lymphocyte markers (indicated by green and pink lines on top of the blow-up Figure) were excluded from the data set. The total 491 genes in the cluster are listed in Supplementary Table S3. Since unbiased between-depots comparison of these genes is not possible they were excluded from subsequent analyses. Clustering was performed on log transformed data by average linkage clustering using Cluster software [37].
(3.83 MB TIF)

Table S1 Spermatozoa were harvested from the epididymal head of mice fed basal chow diet. Fatty acid content is given as grams of fatty acid per 100 grams of fatty acid methyl ester. SD is standard deviation. N -5.
(0.04 MB DOC)

Table S2 Animal weight at the onset of high-fat diet.
(0.05 MB DOC)

Table S3 List of members in cluster containing lymphocyte specific genes. The cluster was identified as described in Supplementary Figure S1.
(0.01 MB XLSX)
