# Supplementary figures and images for: A Combined Transcriptomics and Lipidomics Analysis of Subcutaneous, Epididymal and Mesenteric Adipose Tissue Reveals Marked Functional Differences
Source: PLoS One. 2010 Jul 12;5(7):e11525. doi: 10.1371/journal.pone.0011525 (PMC2902507; doi:10.1371/journal.pone.0011525)

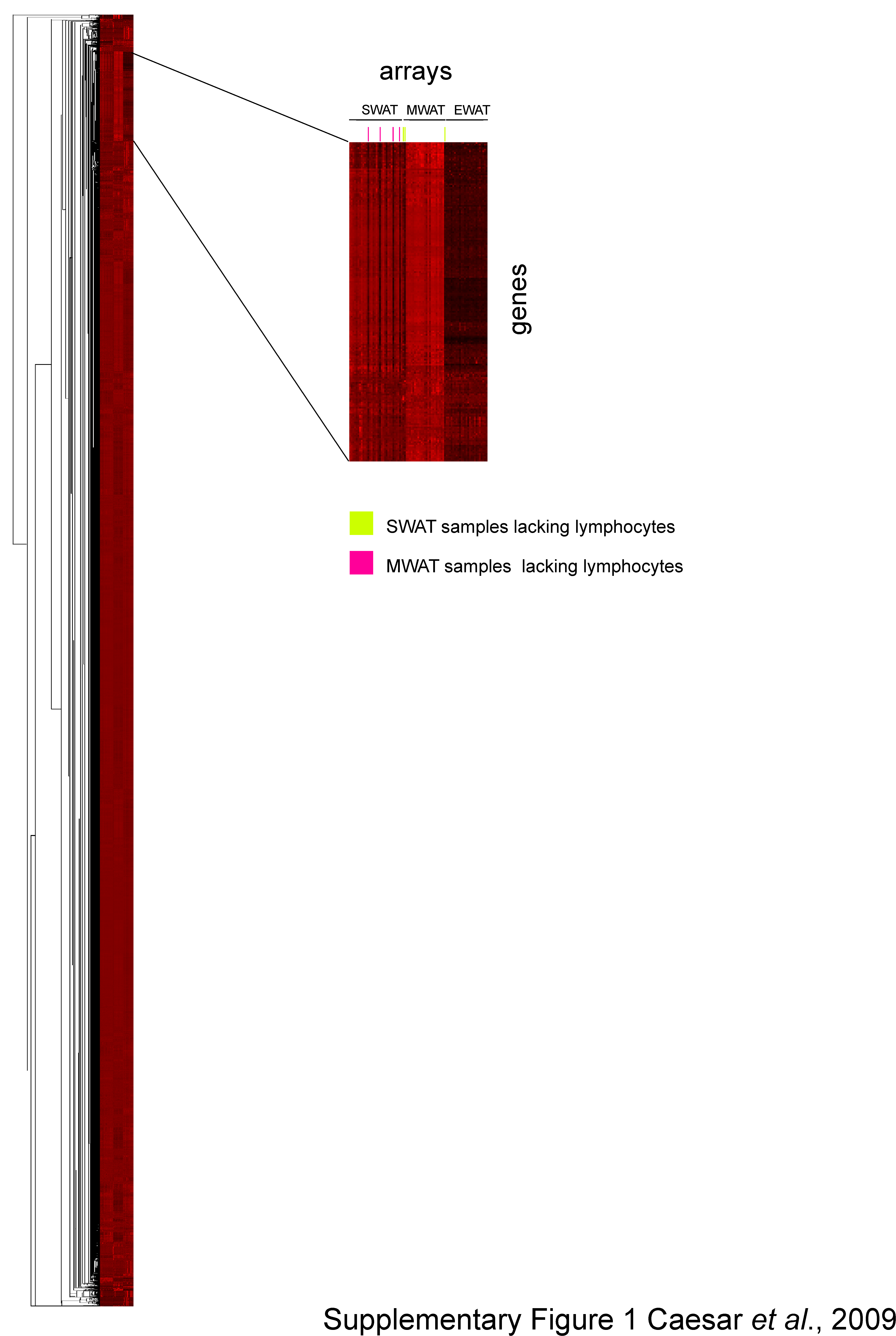

Supplement: Figure S1 — Hierarchical clustering reveals lymphocyte specific genes in adipose tissue samples. Lymphocyte specific genes are present in some adipose tissue samples but not in others. A cluster with a correlation coefficient of 0.97 containing lymphocyte specific genes was identified by hierarchical clustering of array data from mesenteric (MWAT), subcutaneous (SWAT) and epididymal (EWAT) adipose tissue samples (dendrogram and heat-map representing the whole data-set shown to the left, blow-up of cluster containing lymphocyte specific genes shown to the right). Within this cluster genes are expressed in 28 of 32 SWAT samples, 23 of 26 MWAT samples and 0 of 25 EWAT samples. The MWAT and SWAT arrays not expressing lymphocyte markers (indicated by green and pink lines on top of the blow-up Figure) were excluded from the data set. The total 491 genes in the cluster are listed in Supplementary Table S3. Since unbiased between-depots comparison of these genes is not possible they were excluded from subsequent analyses. Clustering was performed on log transformed data by average linkage clustering using Cluster software [37]. (3.83 MB TIF) [file pone.0011525.s001.tif]
