# Supplementary material for: A Combined Transcriptomics and Lipidomics Analysis of Subcutaneous, Epididymal and Mesenteric Adipose Tissue Reveals Marked Functional Differences
Source: PLoS One. 2010 Jul 12;5(7):e11525. doi: 10.1371/journal.pone.0011525 (PMC2902507; doi:10.1371/journal.pone.0011525)
Supplement: Table S1 — Spermatozoa were harvested from the epididymal head of mice fed basal chow diet. Fatty acid content is given as grams of fatty acid per 100 grams of fatty acid methyl ester. SD is standard deviation. N -5. (0.04 MB DOC) [file pone.0011525.s002.doc]

| **Fatty acid** | **g/100g** | **SD** |
| --- | --- | --- |
| C14:0 | 0.43 | 0.12 |
| C15:0 | 0.10 | 0.03 |
| C16:0 | 11.96 | 1.61 |
| C16:1 | 1.86 | 1.06 |
| C17:0 | 0.30 | 0.06 |
| C18:0 | 20.68 | 3.95 |
| C18:1,t6-11 | 0.27 | 0.08 |
| C18:1,c9 | 13.24 | 2.73 |
| C18:1,c11 | 1.34 | 0.17 |
| C18:2, ω-6 | 9.63 | 3.33 |
| C20:0 | 0.17 | 0.01 |
| C18:3, ω-6 | 0.28 | 0.08 |
| C18:3, ω-3 | 0.35 | 0.28 |
| C20:1, ω-9 | 0.18 | 0.04 |
| C20:2, ω-6 | 0.24 | 0.01 |
| C22:0 | 0.54 | 0.15 |
| C20:3, ω-6 | 0.71 | 0.09 |
| C20:4, ω-6 | 18.12 | 3.99 |
| C23:0 | 0.12 | 0.02 |
| C20:5, ω-3 | 0.67 | 0.22 |
| C24:0 | 0.73 | 0.21 |
| C24:1, ω-9 | 0.31 | 0.07 |
| C22:4, ω-6 | 0.14 | 0.02 |
| C22:5, ω-6 | 1.53 | 0.36 |
| C22:5, ω-3 | 0.37 | 0.03 |
| C22:6, ω-3 | 7.37 | 1.08 |
| Unknown | 8.35 | 0.55 |

**Supplementary Table 1. Fatty acid composition of spermatozoa isolated from the epididymal head of ApoE3Leiden mice.** Spermatozoa were harvested from the epididymal head of mice fed basal chow diet. Fatty acid content is given as grams of fatty acid per 100 grams of fatty acid methyl ester. SD is standard deviation. N - 5.
