# Supplementary material for: A Combined Transcriptomics and Lipidomics Analysis of Subcutaneous, Epididymal and Mesenteric Adipose Tissue Reveals Marked Functional Differences
Source: PLoS One. 2010 Jul 12;5(7):e11525. doi: 10.1371/journal.pone.0011525 (PMC2902507; doi:10.1371/journal.pone.0011525)
Supplement: Table S2 — Animal weight at the onset of high-fat diet. (0.05 MB DOC) [file pone.0011525.s003.doc]

| Animal weight at onset of high-fat diet (g) | | | | |  |
| --- | --- | --- | --- | --- | --- |
| 0 | 1 | 6 | 9 | 12 | Time-point of sacrifice (weeks) |
| 26.9 | 27.1 | 22.8 | 26.6 | 29.3 |  |
| 33.2 | 27.9 | 27.6 | 29.6 | 29.2 |  |
| 30.6 | 32.6 | 29.4 | 31.7 | 28.5 |  |
| 27.5 | 32.3 | 29.3 | 29.4 | 26.3 |  |
| 33.3 | 26.6 | 29.5 | 31.1 | 28.2 |  |
| 31.7 | 28.6 | 28.5 | 29.4 | 31.8 |  |
| 29.5 | 24.8 | 33 | 29.3 | 30.2 |  |
| 25.9 | 33.2 | 34 | 30.4 | 26.3 |  |
| 29.6 | 29.7 | 29.3 | 28 | 31.3 |  |
| 28.7 | 30.4 | 31.2 | 27.8 | 31 |  |
| 26.9 | 34.1 | 29.4 | 25.2 | 28.1 |  |
| 29.8 | 29.1 | 33.5 | 28.2 | 31.1 |  |
| 27.8 | 28.3 | 30.5 | 32 | 33.6 |  |
| 31.2 | 27.3 | 30.8 | 29.2 | 30.1 |  |
| 32 | 29.5 | 32.3 | 27 | 31 |  |
| 30.4 | 29.9 |  |  | 32.2 |  |

**Supplementary Table 2. Animal weight at the onset of high-fat diet**.
